# Action Priority: Early Neurophysiological Interaction of Conceptual and Motor Representations

**DOI:** 10.1371/journal.pone.0165882

**Published:** 2016-12-14

**Authors:** Dirk Koester, Thomas Schack

**Affiliations:** 1 Neurocognition and Action Research Group–Biomechanics, Faculty of Psychology and Sport Science, Bielefeld University, Bielefeld, Germany; 2 Cluster of Excellence–Cognitive Interaction Technology (CITEC), Bielefeld University, Bielefeld, Germany; 3 Research Institute for Cognition and Robotics (CoR lab), Bielefeld University, Bielefeld, Germany; University of Nottingham, UNITED KINGDOM

## Abstract

Handling our everyday life, we often react manually to verbal requests or instruction, but the functional interrelations of motor control and language are not fully understood yet, especially their neurophysiological basis. Here, we investigated whether specific motor representations for grip types interact neurophysiologically with conceptual information, that is, when reading nouns. Participants performed lexical decisions and, for words, executed a grasp-and-lift task on objects of different sizes involving precision or power grips while the electroencephalogram was recorded. Nouns could denote objects that require either a precision or a power grip and could, thus, be (in)congruent with the performed grasp. In a control block, participants pointed at the objects instead of grasping them. The main result revealed an event-related potential (ERP) interaction of grip type and conceptual information which was not present for pointing. Incongruent compared to congruent conditions elicited an increased positivity (100–200 ms after noun onset). Grip type effects were obtained in response-locked analyses of the grasping ERPs (100–300 ms at left anterior electrodes). These findings attest that grip type and conceptual information are functionally related when planning a grasping action but such an interaction could not be detected for pointing. Generally, the results suggest that control of behaviour can be modulated by task demands; conceptual noun information (i.e., associated action knowledge) may gain processing priority if the task requires a complex motor response.

## Introduction

The human hand is an important tool to interact with our surroundings. We often use our hands to explore and manipulate objects or to reach for, grasp and place objects. Such actions often require very precise motor control, for example when drinking hot tea from a mug or when handling multiple objects at the same time. The hand can also be very powerful when holding heavy objects or providing body support (e.g., holding on tight on the bus or in sports when making a handstand or climbing [1]). Accordingly, Napier [2] introduced a major distinction among grip types, namely, precision and power grips. But, hands are not only effective for physical interactions. They can also be used for social, communicative and even linguistic functions (i.e., waving, gesturing or sign language, e.g., [3,4]) and these functions may have co-evolved ([5,6]). That is, the human hand can be an end effector of the language and the motor control system [7].

Here, we are interested in voluntary actions, specifically in uni manual grasping, without denying the importance of sensorimotor processes (e.g., for force regulation to avoid object slipping [8,9]). Such manual movements proceed, generally, in two stages as described in the two component model which dates back to Woodworth [10,11,12]. This model distinguishes a planning and a control phase; the former is assumed to be pre-programmed and the latter under feedback control. The planning component is more susceptible to perceptual and cognitive processes whereas the control component is more closely related to executing and monitoring motor commands, cf. [13,14] for review. Besides, voluntary grasping movements are determined by object features, e.g., [15] *and* by action plans [16] and goals, i.e., cognitive processes and anticipated future states, e.g., [17,18]. Human voluntary actions are oftentimes guided by verbal processes as in instructions or requests. That is, some form of interactive processing between the motor control system and the language system seem to be necessary but the precise nature of the underlying neurophysiological interaction is not fully understood.

Various frameworks have been proposed on the relationship between language and action, e.g., [19,20,21] for recent reviews. On the one hand, there are strictly symbolic accounts which assume an amodel, central processing system for language that is functionally independent from other cognitive domains. On the other hand, the embodiment framework assumes that sensory object properties and action features pertaining to the same object share some representational aspects with abstract, symbolic representations for objects and actions, specifically, nouns and verbs, respectively, e.g., [22,21,23]. Accordingly, functional interactions among the domains of motor control and symbolic word representations can be expected ([24,25] for similarities in the structures of sentence representations and action sequences).

One can distinguish strong and weak versions of the embodiment approach, cf. [20]. While strong versions argue that cognitive processes are completely grounded in sensory-motor systems, weak versions assume that language (usually restricted to comprehension) draws on both abstract, symbolic and embodied representations. There is accumulating evidence for embodied processing of verbs (actions) and concrete nouns (objects) in support of weak embodiment views [20]. In contrast, the processing of abstract concepts and metaphorical actions are less well-understood and the appropriateness of the embodiment view is still discussed. Another aspect that is hardly understood according to Horchak et al [20] is “deep comprehension,” that is, the activation of inferred knowledge. For example, whether and how associated knowledge of nouns such as associated actions is activated (by inference or association) is not yet fully understood. (As a side note, formally, actions are not part of a noun’s definition, that is, its semantics; actions are commonly expressed by verbs. Thus, the question arises whether and how action knowledge can be activated by noun representations?) The embodiment view serves as a theoretical framework for our project.

The aim of the present study was to test the time course of the potential interplay of symbolic, conceptual (word) representations and concrete, motor commands (part of grip representations) to better understand the functional interplay of language and action processes. Such insights might help to understand whether and when the task has a modulating influence on behaviour. Participants were asked to read letter strings and, for words, they had to execute either a power or a precision grip. The words (nouns) could denote larger or smaller objects that require either a power or a precision grip if grasped. Hence, the conceptual information conveyed by the nouns was implicit and could be (in)congruent with the grasping action that had to be performed. We recorded event-related brain potentials (ERPs) to determine the time course of such a neurophysiological interplay and to evaluate the role of conceptual information.

While there is an on-going discussion about the different sub functions of the neural basis of grasping and their relations [26], there emerges consensus regarding a parieto-frontal network as the neural underpinning of grasping with evidence from different populations and methodologies, e.g., [18,27,28,15,29,30,31] but see also [32]. ERPs are well-suited for and have recently been applied successfully to the examination of overt movements [33,34,35,36,37]. Thus, ERPs with their high temporal resolution can be used to investigate fast neurocognitive processes of language comprehension and grasp planning/execution. Moreover, ERPs can be time-locked to the presentation of stimuli or to the (onset of the) movement (response-locked ERPs). Stimulus-locked ERPs capture in principle perceptual and cognitive brain processes (in the sense of stimulus processing or evaluation) whereas response-locked ERPs reflect motor-related brain processes, e.g., [38,39]. Hence, stimulus- and response-locked ERPs may conceptually be close to the planning/execution distinction related to the two component model of voluntary manual movements. To the best of our knowledge, the present ERP study is the first to investigate noun reading and its associated action knowledge together with the execution of different grasp types.

Regarding action execution, van Elk and colleagues [40] related recently the N400 ERP component to action execution. These authors compared meaningful and meaningless actions by showing pictures of a cup or a magnifying glass. Participants had to perform a grasp-and-transport action (bringing a cup or a magnifying glass to the mouth or to the eye). Meaningful actions elicited an increased N400 amplitude (380–450 ms after stimulus onset) which was interpreted to reflect the retrieval of semantic action information [40]. De Sanctis and colleagues [37] reported a sustained, N400-like effect for grasping small objects (precision grip) compared to grasping large objects (whole hand grip). This N400-like effect was observed for the time window 400 to 800 ms (beginning already at 300 ms) after stimulus onset. De Sanctis et al. [37] were interested in the kinematics of reach-to-grasp movement which was recorded in addition to the EEG signal. Since the ERP analyses were stimulus-locked, the N400-like effect was interpreted as to reflect the *planning* of the motor action, that is, the cognitive processing aspects. In order to investigate the ERP correlates of motor aspects specifically, response-locked ERPs would be needed but were not reported. Hence, it remains still difficult to distinguish between action planning and execution with regard to those ERP measures. In contrast, response-locked ERP analyses of grasping movements have yielded slow waves at frontal and posterior electrodes, e.g., [33,41,42] or modulations of the Bereitschaftspotential (BP [35,43]) which also varied between frontal and posterior electrodes but these grasping studies did not consider additionally language processes. After all, the time window of the reported N400-like effect indicating grasp-related information processing is, generally, in line with the extended durations of slow waves in other ERP studies on grasping [33,35,41,42].

Note that the N400 is also well-investigated in the language domain where it has been related to meaning processing, e.g., [44,45,46]. Such N400 effects for language processing for single word processing have been reported in various time ranges and last often up to 650 ms or longer, especially in action contexts, e.g., [47,34,44] which resemble the above-mentioned time ranges for grasping-related N400 effects. The N400 can begin as early as 200 ms after word onset (as a lower limit [48]).

The integrated processing of language meaning and motor control processes has been investigated neurophysiologically, cf. [49,50] and behaviourally, cf. [51,52]. For example, action-irrelevant words referring to objects of different sizes (e.g., “apple” or “needle”) influence the hand shape (maximum grip aperture) when grasping unrelated objects (e.g., wooden blocks, cf. [53,13,54]. Similarly, Lindemann and colleagues [55] showed that reading words such as “mouth” or “eye” facilitated grasping execution when the words matched the to-be-grasped object (cup or magnifying glass, respectively; see also [56]).

Neurophysiologically, co-activations of the primary and somatosensory motor cortex by reading were reported very early in the time range of 100 and 200 ms after word onset [49,50] in line with the embodiment framework as stated by these authors. Similarly, Boulenger et al. [57] compared the reading of verbs, nouns and consonant strings and whether the reading impacts the ERPs and the kinematics of reach-and-grasp movements. Interestingly, not only wrist acceleration was affected by word class, but also the ERP amplitudes differed for verbs and nouns during the first 350 ms after word onset. This word class effect was interpreted as a functional distinction that the brain makes due to the action information of verbs. However, reading consonant strings led to a similar ERP response as reading verbs. But, consonant strings do not, of course, carry action-related information, that is, they have no (action) semantics and should thus rather pattern together with nouns and not with verbs which do refer to actions; this aspect remains unresolved at present. Furthermore, Proverbio and colleagues [58] reported access to object affordances (of tool pictures) to take place in the first 250 ms even though the functional properties of the objects were not task-relevant. But note, that the above-mentioned ERP studies all analysed the ERP signal in a stimulus-locked but not a response-locked manner. Thus, these studies are limited regarding their implications as to the planning/execution distinction of (voluntary) manual actions. The early time of occurrence suggests that relevant information for the current behaviour, whether conveyed by language or pictorial information, can be processed well before conceptual information as indicated by N400 effects. That is, it seems to be possible (and worth testing) that a neurophysiological interaction of language and motor control processes occurs already between 100 and 200 ms after word onset which would imply the availability of language *and* action relevant information. Note that movement adjustments during the first few hundred ms have been reported for manual actions (grasping and reaching [59,60]).

In contrast, Amsel et al. [61] argue that action-related information (graspability) becomes available only *after* conceptual information (semantic category: living vs. non-living) has been processed. In a go/no-go paradigm, participants had to judge semantic category membership and graspability of written nouns via button-press responses. The ERPs suggested that conceptual information was processed about 150–200 ms after word onset but graspability affected the ERP after about 340 ms, i.e., about 190 ms after conceptual information. Hence, action-related noun information was argued to be secondary to conceptual information.

Given the nature of the task (noun reading/evaluation), Amsel et al. [61], p.9 discuss whether conceptual information processing is *obligatory* before action-related information becomes available. Actually, these authors suggest that the timing of access to conceptual and action-related information could be variable because processing of conceptual and action-related information may be situation-specific. That is, their task may have set priority to conceptual information processing due to the reading requirement but did not call for a specific, complex manual action for which the word would be critical (see also [62]). (Note that graspability and semantic category information were both conveyed by the words alone in the Amsel et al. study.) Consequently, we hypothesised that if the reading material were directly related to an action response, for example, grasping with a specific grip type, the processing priority may change and, hence, the timing of the neurophysiological processing. In such a situation, action-related word information may be processed as early as conceptual information [58,50,57] and in an integrative manner as would be suggested by embodiment frameworks (for task influence on manual responses see also [63]).

In the present study, we created a situation in which the words are directly relevant to a complex, manual response (grasping) even if the words served only as imperative signals. Here, we investigated the neurophysiological interaction of symbolically coded, conceptual information (part of lexical representations) with concrete, motor commands (part of grip type representations) when responding manually. Participants were asked to perform a go/no-go lexical decision task on letter strings. In response to words, participants had to grasp a cubic object that required either a precision or a power grip while the electroencephalogram (EEG) was recorded. The nouns denoted either objects that would be grasped with a power (e.g., “apple”) or a precision grip (e.g., “raisin”). That is, the implied conceptual grasping information could be congruent or incongruent with the grip type that had to be executed. For pseudo words, participants withheld their response. Furthermore, we asked if an interaction of conceptual information and motor commands (for grip types) would be specific for grasping by introducing a control block in which participants pointed at the objects instead of grasping them. The control block was intended to provide a qualitative comparison to the grasping block. Even though grasping and pointing movements differ kinematically, we surmised that, the similar (neurophysiological) effects should be observed for pointing as for grasping if the potential effects are unspecific (e.g., if they are functionally related to reaching). However, if the effects are specific for grasping, they should not be found in the pointing block.

Based on findings of integrated processing of language and motor control, e.g., [23,57] and the findings that nouns can affect manual responses, e.g., [53,55], we expect also neurophysiologically an integrated processing of conceptual word information and motor commands during grasping behaviour. Such an integrated processing can be expected to arise in the planning phase of manual action as it has been shown that uni manual grasping interferes with verbal working memory during the planning rather than during the control component [64]. From an ecological point of view, optimal behaviour has priority [65,66]. Hence, action-related conceptual information may be processed with priority if the situation requires complex actions such as grasping. That is, it should be processed earlier in an action situation than in reading situations. Adapting the cognitive processing to the current task demands might be beneficial to behavioural control.

According to the theoretical view that planning and online control are distinct stages of motor actions, e.g., [12,10], we expected a main effect of conceptual information for reaction times (RTs) as the written words served as the imperative signal [67]. An interaction of conceptual noun information and motor commands (i.e., grip type) would be of interest but previous related work did not report similar interactions in RTs [68,57,61]. In contrast to RTs, movement times (MTs) should reflect the grip requirements, i.e., a grip type effect.

Importantly, we predicted an interaction of conceptual information and grip type in the ERP amplitudes, if motor control and the language system are functionally related [7]. That is, congruent noun grip combinations should differ from incongruent noun grip combinations. Such interactions are expected in the stimulus-locked ERPs which reflect perceptual/cognitive processing. If such an interaction effect is unspecific for grasping, we should also find this effect also in the pointing task (control block). If, however, it is specific for grasping, no interaction is expected in pointing. If behavioural control (the integrated processing of language and motor control) is *not* situation-specific, action-related, conceptual information should affect the ERP only in the time range of the N400, after about 350 ms [40,61]. In contrast, if behavioural control is situation-specific, we expect an earlier influence of action-related conceptual information on the ERP than in the study of Amsel et al. [61], in particular between 100 and 200 ms [50,49,57].

Furthermore, regarding motor stage processing (related to the online control component), the response-locked ERPs will be explored as we are not aware of according precursory studies. A main effect of grip type can be expected, at least after movement initiation because voluntary grip type executions should be controlled by partly differing central nervous activity. The response-locked ERPs may also reveal differences in the Bereitschaftspotential (or similar slow potential shifts), for example, if motor preparation would differ among conditions, cf. [35]. (A related motor component is the contingent negative variation; CNV [69]. However, a CNV is usually obtained in an S1-S2 paradigm with S2 being an imperative signal. Here, we did not use an S1-S2 paradigm and the letter strings were not imperative signals as pseudo words did not require a response.) If there would be an interaction of conceptual noun and grip type information, these response-locked ERPs would provide evidence for an integrated processing of these information in the online control component.

## Materials and Methods

### Participants

Twenty-eight, right-handed native speakers of German participated for course credit or monetary compensation. Two participants (m) had to be excluded because too few trials remained for response-locked ERPs after correction for movement artefacts. The remaining 26 participants (11 male; average lateralization coefficient 96.7; [70]) were on average 24.7 years of age (range 20–30). All participants had normal or corrected-to-normal visual and auditory acuity. Participants gave written informed consent, and the experimental procedure was approved by the ethics committee at Bielefeld University and adhered to the ethical standards of the latest revision of the Declaration of Helsinki [71].

### Design

The experiment consisted of two blocks; the main experimental block (grasp execution) and a control block (pointing). Both blocks used a 2×2 within-subjects design. The experimental factors were *action-related conceptual information of the noun* (*noun concept* for short: small vs. large nouns) and *grip type* (precision vs. power grip) in the grasping block. In the pointing block, the factors were *noun concept* and *object size* (small vs. large; for pointing). The order of blocks was counter balanced across participants. Regional factors (anterior-posterior & left-right) were also included; see below section 2.6). Action type (grasping vs. pointing) was not included because the two blocks do not constitute independent levels of one variable, e.g., [72,73,74]. Furthermore, grasping and pointing movement are not strictly comparable as they differ kinematically [75,59,76] and seem to rely on non-identical neural networks [31]. The dependent variables were reaction times (RT), movement times (MT) and mean amplitudes of the ERPs. RTs (release button response) were measured relative to the noun onset. MTs were defined as the duration from start button release to object lift.

### Stimuli and set-up

Thirty words (concrete object nouns) were collected for each class of implied action (e.g., “apple” for nouns implying power grips [henceforth “large” nouns] and “raisin” for precision grips [“small” nouns]) as critical items (60 in total). Both word lists were matched for word frequency (lemma frequency according to Celex database [77]), length (number of letters), number of syllables and number of morphemes, proportion of syntactic genders, bigram and trigram frequencies (according to *dlexDB* database; http://www.dlexdb.de/ [78]) and concreteness/imagebility (cf. Table 1). As filler items, 30 abstract nouns (e.g., “guaranty”) and 15 pseudo words were selected. Abstract nouns served as distraction from graspability of the target words (go trials). Pseudo words were created by replacing one vowel of the target words leaving the word’s syllable structure intact to closely parallel the word items; all pseudo words adhered to German phonology. Pseudo words were employed to realise the go/no-go task and to ensure attentive processing of the visual stimuli, i.e., the active comprehension of the critical words. The total list contained 57% critical words, 28.5% abstract words and 14.5% pseudo words (see below). Critical words required in 50% a precision and in the other 50% a power grip.

**Table 1:** Stimulus characteristics for nouns denoting small or large objects. Concreteness was rated on a Likert scale (ranging from 1 to 7; abstract, i.e., filler nouns were rated 3.5). Proportions of syntactic genders are given for feminine / masculine / neuter classes. Stimuli did not differ statistically in any of these variables.

*Noun concept*	*Freq / 1 mill*.	*Let*	*Syll*	*Morph*	*Gender*	*Bigram freq*	*Trigram freq*	*Concrete-ness*
*Small*	38.2	7.1	2.3	1.3	0.6/0.3/0.1	279769	141548	6.8
*Large*	55.5	6.4	2.2	1.2	0.6/0.3/0.1	278462	155623	6.7

Note: Freq-lemma frequency; Let-number of letters; Syll-number of syllables; Morph-number of morphemes.

The objects for the grasping task were three cubes (length of the edge 25, 50 & 80 mm) with rounded corners (corner radius 7.5, 15, & 25 mm, respectively). All objects were of the same weight (100 g) and colour (black). The smallest cube requires a (two finger) precision grip whereas the largest cube requires a power grip for grasping (due to the object size and by instruction). The medium sized cube could be grasped in more than one way and served as a filler object for the action task.

**Fig 1:**
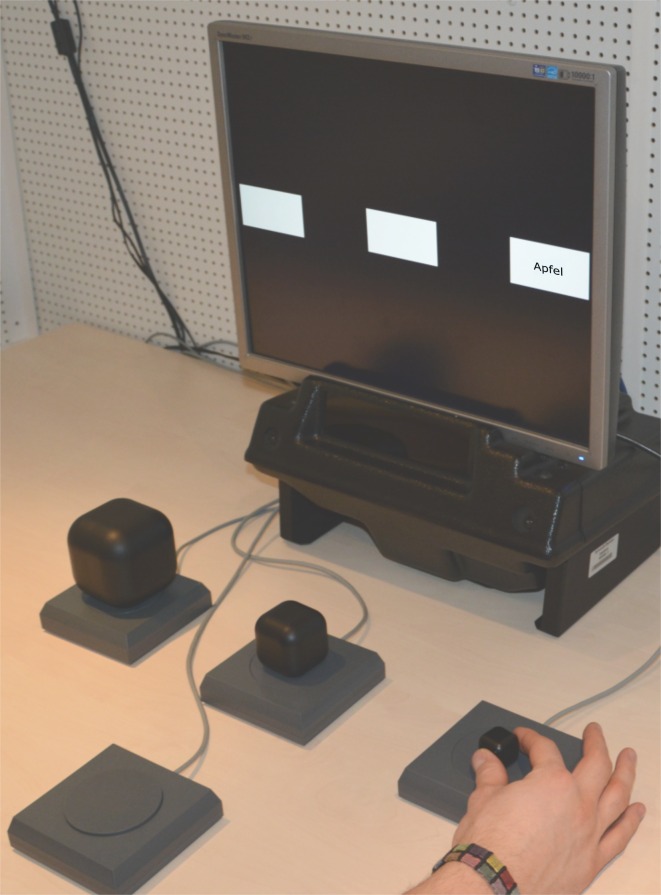
Setup. The experimental setup, incl. the monitor indicating the boxed areas for the stimulus presentation, the three objects on pressure-sensitive buttons and a start button (closest to participant). All object positions could be reached comfortably with the extended arm. The object positions of the large and the small object were counter balanced.

Participants were seated 70–80 cm in front of a monitor. Three squared push buttons (11 cm length of the edge & 2 cm height with a circular depressible area of 8 cm in diameter) were placed 20 cm in front of the monitor with 3 cm space in between. The target objects were placed on these buttons. A fourth push button was placed between the middle target object button and the participant so that the participant could comfortably press and hold down this start button with her right hand. The whole set-up which is shown in Fig 1 was aligned so that the participant could easily grasp and lift the objects with their right hand without changing her reclined body posture. The three target buttons were, furthermore, aligned with three grey (rectangular) target areas that would later appear on the screen. The target areas indicated which object had to be grasped and lifted when a word appeared in one of these.

Each participant received a different trial order. Each critical word was presented once at the position of the small and once at the position of the large object. Ten of the critical words (varied across participants) were also presented at the medium sized object. Abstract and pseudo words were assigned and distributed in the same ratio. For the two grasping blocks the letter string-object assignment was reversed. The trial order was pseudo randomised, i.e., letter strings were not immediately repeated and the correct response (position) would not be repeated more than four times.

### Procedure

**Fig 2:**
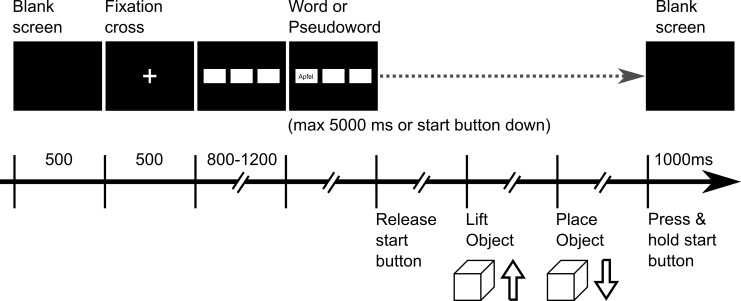
Trial procedure. Schematic representations of the trial procedure (similar for grasping and pointing). Participants had to grasp and briefly lift the (spatially) associated object for word stimuli using a precision or a power grip depending on the object size. Pseudo word constituted no-go trials in which the start button should not have been released. In the pointing block, grasping and lifting was replaced by pointing at the associated object (with an open hand).

Participants were seated in a dimly lit, sound-attenuated and electrically shielded booth. They were instructed to read carefully the stimuli while pressing the start button and respond to (correctly spelled) German words by releasing the start button, grasping the spatially aligned object, lift it for about 1–2 seconds, replacing the object and returning to (and pressing) the start button. For pseudo words, participants had to withhold their response (no-go), i.e., to keep the start button down until the next letter string appears. The procedure is illustrated in Fig 2.

The experiment consisted of a practise block (12 trials which were not used during the experiment proper), two grasping blocks and one pointing block; pointing had to be done with the fully opened hand (no spacing between fingers). The order of grasping and pointing was counterbalanced across participants, and within the two grasping blocks, the position of the small and the large object (left vs right) was also counterbalanced across participants; half of the participants would start the grasping with the small object on the left and the other half with the small object on the right position. There was a short debriefing session after the experiment. The whole session lasted approximately 90 min.

The experiment started when the participant pressed the start button. After 500 ms (blank screen) a fixation cross was shown at the centre of the screen for 500 ms that was then replaced by three horizontally aligned rectangular white areas (100×200 pixel on a black background) at medium height of the screen. After a variable interval of 800–1200 ms a letter string appeared in one of the three areas. For word stimuli, participants had to grasp and lift the assigned object, replace it after about 1 to 2 sec. and then press and hold the start button until the next trial began. The inter trial interval was 1,000 ms. No feedback was provided. Stimulus presentation was controlled by the Presentation software (version 14.4, http://www.neuro-bs.com/).

### EEG Recordings

The EEG was recorded using a 64 channel amplifier (ANT Neuro). The EEG cap was equipped with Ag/AgCl electrodes placed according to the 10–10 system [79]. The EEG was low-pass filtered (DC-138 Hz), digitised (512 Hz sample rate) and electronically stored. To control for eye movements bipolar horizontal and vertical electrooculograms (EOG) were recorded. Electrode impedance was kept below 5 kΩ and the common reference was used during recording.

### Data analysis

The EEGLAB toolbox [80] for MATLAB (version 7.6; http://www.mathworks.co.uk/) was used for EEG data analysis. The data were band-pass filtered offline (0.1–30 Hz) and re-referenced to the average mastoid activity. Ocular artefacts were corrected using the correction procedure of Gratton, Coles, and Donchin [81]. For automatic rejection a moving window approach was used (200 ms extension with a threshold ± 50 μV) and epochs were visually double-checked. Incorrect responses and epochs that still contained (eye) movements were also excluded. This procedure resulted in the exclusion of 10.5% of the trials for the stimulus-locked and 17.0% for the response-locked ERP analyses. All experimental conditions were affected equally.

The stimulus-locked ERPs were time-locked to the letter string presentation with a 100 ms baseline before letter string onset. For response-locked analyses, ERPs were calculated relative to movement onset (i.e., start-button release) with a baseline from -1,300 to -1,100 ms. This baseline was defined to precede the presentation of the letter strings, cf. [82]. (Note that the response-locked ERPs did not depend on the specific baseline; an earlier baseline (-2,400 to -2,200 ms) yielded the same ERP pattern.) Response-locked analyses were performed in order to explore further the neurophysiological brain response for the current dual task and, therefore, time windows for response-locked analyses were based on visual inspection but used the same regions of interest (ROIs; see below) as for the stimulus-locked analyses. Additionally, the response-locked ERPs of the electrodes C3 (left) and C4 (right) were also tested as typical indicators of activity from (the vicinity of) the motor cortex, cf. [83,84,85]. Following Hauk et al. [49] Penolazzi et al. [50] and Boulenger et al. [57], the time window from 100 to 200 ms (stimulus-locked) was defined for testing an early interaction effect of *noun concept* and *grip type*, and based on Bach et al. [47], Koester & Schiller [34] and De Sanctis et al.[37], the time window from 500 to 650 ms was defined for testing the N400, that is, a late interaction effect (or N400 main effects). Response-locked time windows were defined on visual inspection of grand ERP averages (exploratory analyses).

The following ROIs were created for the analyses (anterior left: [F5, F3, F1, FC3, FC1]; anterior right: [F6, F4, F2, FC4, FC2]; posterior left: [PO5, PO3 P3, P1, O1]; posterior right: [PO6, PO4, P4, P2, O2]). The ROIs were arranged in relation to the frontal and posterior ERP effects in previous work [33,36,41,42,37]. Average ERP amplitudes were calculated separately for each ROI and for each experimental condition. All statistical analyses were performed using the software R [86].

## Results

### Reaction & movement times

Participants showed no difficulties with the experimental task. In the grasping block, the two-way ANOVA with the factors *noun concept* and *grip type* on the log mean reaction times (RTs) yielded a main effect of *noun concept* (*F*_1,25_ = 4.19; *p* = .05; *Ω*^*2*^ *=* 0.0037). Neither the main effect of *grip type* nor the interaction of these two factors was significant (both *F*s_1,25_ < 1.13; ns). Nouns associated with a power grip (“large” nouns) led to faster reactions than nouns with a precision grip association (“small” nouns; 10 ms difference). The same ANOVA on the log means of movement times (MTs) yielded only a main effect of *grip type* (*F*_1,25_ = 72.04; *p* < .0001; *Ω*^*2*^ *=* 0.6511). Neither the main effect of *noun concept* nor the interaction of both factors were significant (*F*_1,25_ < 1; ns). Power grips were executed faster than the precision grips (93 ms difference). For the according condition-specific values see Table 2.

**Table 2:** Grasping: mean reaction times (RT) and movement times (MT) in ms per experimental condition and the 95% confidence intervals.

**RT**	*Grip type*		
*Noun concept*	*Power*	*Precision*	*Mean*
*Large*	**844** [835, 854]	**835** [827, 844]	**840** [835, 845]
*Small*	**850** [841, 860]	**850** [841, 859]	**850** [845, 855]
*Mean*	**847** [841, 854]	**843** [836, 849]	
**MT**			
*Large*	**642** [620, 655]	**737** [725, 749]	**688** [684, 692]
*Small*	**647** [638, 657]	**737** [725, 749]	**691** [687, 694]
*Mean*	**644** [635, 655]	**737** [726, 748]	

In the control block (pointing), RTs were also analysed by a two-way ANOVA with the factors *noun concept* and *object size* (the latter corresponding to grip type in the grasping block). One participant’s data had to be excluded as the mean RTs in this block in one condition were outside three standard deviations of the condition-specific group means. (The effects for RTs and MTs of the grasping block did not change when excluding this participant for reasons of comparability.) Here, *noun concept* modulated the RTs (*F*_1,24_ = 5.95; *p* < .05; *Ω*^*2*^ *=* 0.0097), but *object size* did not influence the RTs (*F*_1,24_ < 1.08; ns). The interaction was also not significant (*F*_1,24_ < 1; ns). Again, large nouns led to faster reactions compared to small nouns (22 ms difference; see Table 3). Please refer also to S1 and S2 Datasets for RT and MT data.

**Table 3:** Pointing (control block): mean reaction times (RT) in ms per experimental condition and the 95% confidence intervals.

RT	*Cube size*		
*Noun concept*	*Large*	*Small*	*Mean*
*Large*	**879** [861, 898]	**861** [841, 882]	**870** [862, 879]
*Small*	**902** [878, 927]	**882** [863, 902]	**892** [883, 901]
*Mean*	**891** [873, 909]	**872** [854, 890]	

### Stimulus-locked ERPs

**Fig 3:**
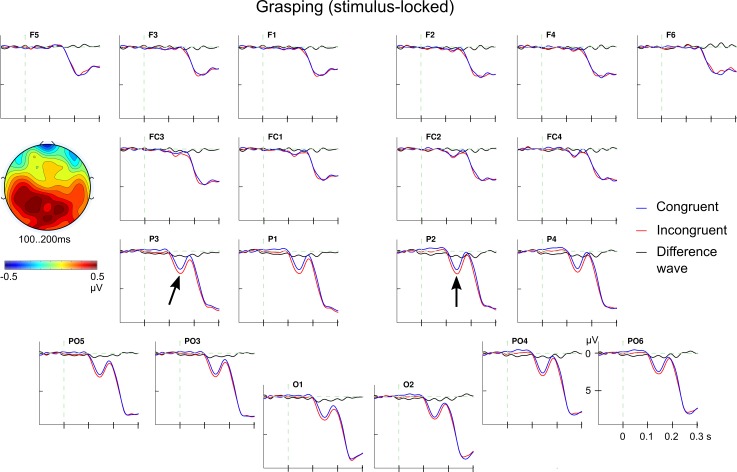
Stimulus-locked ERP effect of (in)congruence in grasping. Grand average ERPs for the effect of (in)congruence between conceptual noun information and grip types (congruent–blue; incongruent–red) and the difference wave (black). The incongruent was more positive than the congruent ERP between 100 and 200 ms. The topographical map (nasion at the top) shows the (posterior) scalp distribution of the effect (incongruent minus congruent). Note that the scale of the maps varies among figures to optimally present the *distribution*, not the size of the effect. Negativity is plotted upwards in this and all subsequent ERP plots.

Regarding the ERPs in the grasping block, the potential interplay of *noun concept* and *grip type* information was tested for the time window 100 to 200 ms after letter string onset with a four-way ANOVA with the factors *noun concept*, *grip type*, left vs. right hemisphere (*LR*) and anterior vs. posterior direction (*AP*). The main effects of *noun concept* and *grip type* were not significant (both *F*s_1,25_ < 1, ns) but the interaction of *noun concept* and *grip type* was significant (*F*_1,25_ = 4.37; *p* < .05; *Ω*^*2*^ *=* 0.001). Furthermore, the three-way interaction of *noun concept*, *grip type* and *AP* was marginally significant (*F*_1,27_ = 3.61; *p* < .1; η^*2*^ *=* 0.0029). (For full within-subjects designs with three or more factors, there is no general agreement how *Ω*^*2*^ should be calculated reliably. As a surrogate generalised η^2^ is reported here.) In follow-up ANOVAs with *noun concept* and *grip type* for the anterior and the posterior ROI, there were no significant effects in the anterior ROI (all *F*s_1,25_ < 2.9; ns). However, the interaction of *noun concept* and *grip type* was significant in the posterior ROI (*F*_1,25_ = 9.01; *p* < .01; *Ω*^*2*^ *=* 0.0059). No other effects were significant in this analysis. A follow-up *t*-test showed that incongruent conceptual noun information (small noun/power grip & large noun/precision grip) led to a more positive ERP between 100 and 200 ms compared with the congruent conditions (small noun/precision grip & large noun/power grip; *t*_25_ = 3.00; *p* < .01; *Ω*^*2*^ *=* 0.0232; cf. Fig 3 & S1 Fig).

**Fig 4:**
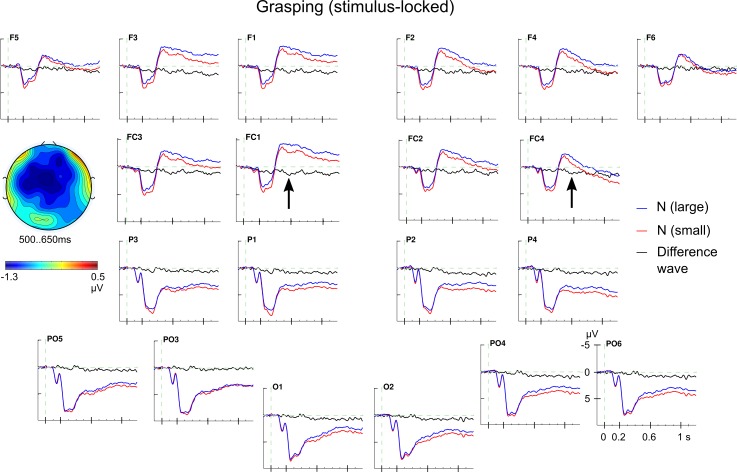
Stimulus-locked ERP effect for noun information in grasping. Grand average ERPs for conceptual noun information. Nouns referring to smaller object (requiring usually precision grips; red lines) showed a reduced N400 effect (500–650 ms) compared with nouns for larger objects (usually grasped with a power grip; blue lines). The difference wave is also shown (in black). The topographical map shows the central scalp distribution of the N400 effect (large minus small). Note, *N (large); N (small)*: conceptual noun information referring to larger or smaller objects.

In order to see whether these effects reflect an early onset of processing, the same four-way ANOVA was performed for the time window 200 to 300 ms post stimulus onset (cf. Fig 4). In this analysis, no main effect nor any interaction involving the experimental factors were significant (all *F*s_1,25_ < 1.6; all *p*s > .23). Only the main effect of *noun concept* (*F*_1,25_ = 2.97; *p* < .1; *Ω*^*2*^ *=* 0.0014) and the *noun × AP* interaction were marginally significant (*F*_1,25_ = 3.08; *p* < .1; *Ω*^*2*^ *=* 0.0004). Separate, follow-up ANOVAs for the anterior and the posterior ROI yielded no significant effects; the main effect of *noun concept* in the anterior ROI missed significance (*F*_1,25_ = 4.15; *p* = .052; *Ω*^*2*^ *=* 0.0036). Importantly, the interactions of *noun concept* and *grip type* were not significant (both *F*s < 1, ns).

Applying the four-way ANOVA to the N400 time window (500–650 ms) yielded a significant main effect of *noun concept* (*F*_1,25_ = 6.04; *p* < .01; *Ω*^*2*^ *=* 0.0093); see Fig 4. Large nouns elicited a more negative ERP amplitude than small nouns, and the effect had a central maximum. The main effect of *grip type* missed significance (*F*_1,25_ = 3.70; *p* = .066; *Ω*^*2*^ *=* 0.0027) and no interaction involving the experimental factors reached significance (all *F*s_1,25_ < 2.04; all *p*s > .16).

**Fig 5:**
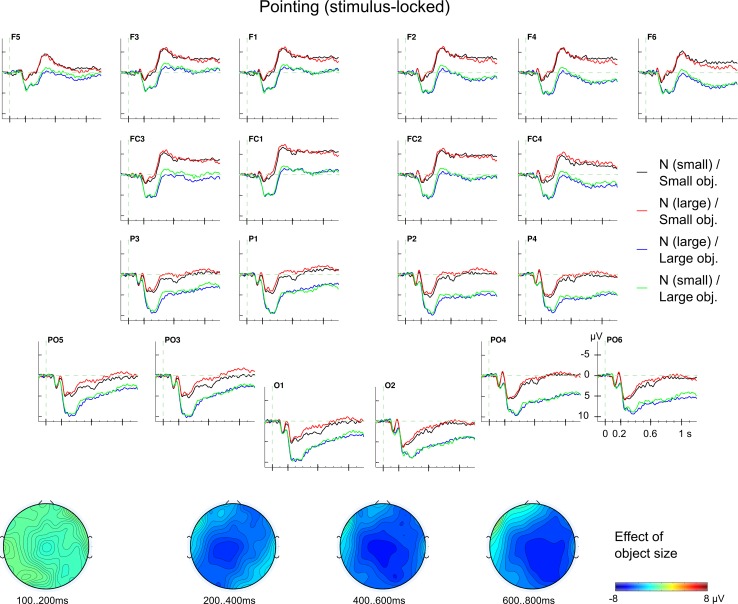
Stimulus-locked ERPs in pointing. Grand average ERPs for the four conditions (*noun concept* × *object size*) in the control block. There was a broadly-distributed, long-lasting effect of object size beginning around 200 ms. The topographical map shows the central scalp distribution of the effect with a late right-hemispheric dominance (small objects minus large objects).

In the control block (pointing), the ERPs (see Fig 5) were characterised by a distinct pattern, i.e., a broadly distributed, sustained, negative slow shift associated with small *object size* (which required precision grips in the grasping block). To analyse this slow shift, an ANOVA with the factors *noun concept*, *object size*, *LR* and *AP* was performed for the time window 200–800 ms. The analysis yielded only a main effect of *object size* (*F*_1,25_ = 13.08; *p* < .01; *Ω*^*2*^ *=* 0.0013). Neither *noun concept* (*F*_1,25_ < 1; ns) nor any interaction involving the experimental factors were significant (all *F*s_1,25_ < 2.08; all *p*s > .16). Descriptively, this object size effect has a centroparietal maximum with a late right-hemispheric dominance (cf. topographical maps in Fig 5).

Importantly, we tested the specificity of the *noun concept-grip type* interaction in the grasping block by analysing the same time window, namely, 100–200 ms in the control block using the same four-way ANOVA (cf. Fig 5) as for the grasping block. Again, only the main effect of *object size* was significant (*F*_1,25_ = 5.72; *p* < .05; *Ω*^*2*^ *=* 0.0250) but *noun concept* was not (*F*_1,25_ < 1; ns). There was also a tendency for *object size* to interact with LR (*F*_1,25_ = 3.87; *p* = .06); no other interaction reached significance (all *F*s_1,25_ < 2.32; all *p*s > .14). We planned for a conceptual (i.e., qualitative) comparison of the ANOVA results for the pointing and the grasping block because the movements differ kinematically [75,59,76]. Including *block* as an additional factor leads to further concerns about statistical power. However, if such a five-way ANOVA (*block × noun concept × grip type × LR × AP*) is performed for the time window 100–200 ms, an interaction of *block* and *grip type* (*F*_1,25_ = 5.579; *p* < .05; *Ω*^*2*^ *=* 0.0076) is found together with a tendency of an interaction for *block × grip type × LR × AP* (*F*_1,25_ = 3.150; *p* = .088; *Ω*^*2*^ *=* 0.0017). (A side remark, consistently, including *block* as a factor in the RT analysis (i.e., in a three-way ANOVA) yields a main effect of block (*F*_1,24_ = 4.329; *p* < .05; *Ω*^*2*^ *=* 0.0044) and of noun concept (*F*_1,24_ = 11.920; *p* < .01; *Ω*^*2*^ *=* 0.0455) supporting our presumption that the two blocks do not elicit strictly comparable movements.) Further exploratory ANOVAs (*block × noun concept × grip type)* for each ROI yielded, next to *block × grip type* interactions main effects for *grip type*, a tendency for a *block × noun concept × grip type* interaction (*F*_1,25_ = 3.169; *p* = .087; η^*2*^ *=* 0.0087) in the posterior left ROI. The suggestive influence of block is in accordance with the well-supported kinematic (and functional) differences between grasping and pointing movements, cf. [75,59,76].

To test the N400 time window (500–650 ms¸pointing block), the same four-way ANOVA was performed on the control block. Again, the main effect of *object size* was significant (*F*_1,25_ = 9.65; *p* < .01; *Ω*^*2*^ *=* 0.0047) but *noun concept* was not (*F*_1,25_ < 1; ns). There was a tendency for an *object size* × *LR* interaction (*F*_1,25_ = 3.58; *p* = .07) and a tendency for a *noun concept × AP* interaction (*F*_1,25_ = 3.50; *p* = .073). No other interaction was significant (all *F*s_1,25_ < 2.4; all *p*s > .11).

### Response-locked ERPs

Response-locked analyses may help to clarify the role of perceptual/cognitive vs. motor processing aspects, especially for the unexpected sustained negativity for the small object in the control block (pointing).

**Fig 6:**
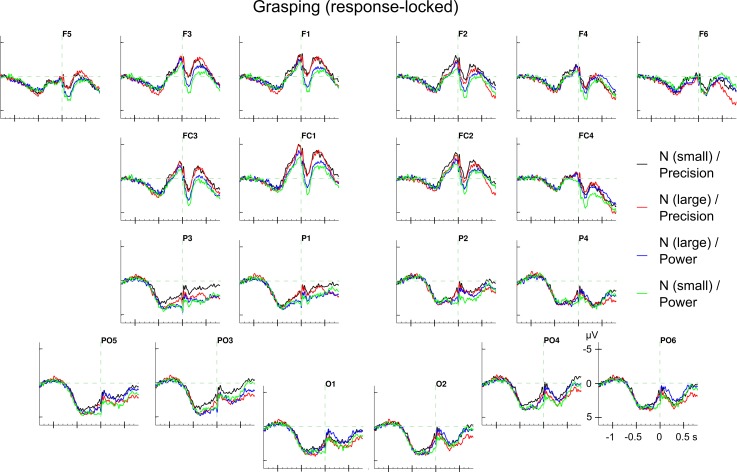
Response-locked ERPs in grasping. Grand average ERPs for the four conditions (*noun concept* × *grip type*). The effect of *grip type* was significant in the left anterior electrodes between 100 and 300 ms.

The response-locked ERPs in the grasping block show a broadly distributed positive shift that begun about one second before movement onset and turned into a negative shift akin to the Bereitschaftspotential (BP [43]) around 500 ms before movement onset. The typical subsequent sequence of pre motor positivity, (PMP; negative going) motor potential (MP) and the movement evoked potential (MEP; extended positivity after movement initiation also known as reafferent potential) begun about 100 to 50 ms before movement onset (see Fig 6; cf. [87,88]).

**Fig 7:**
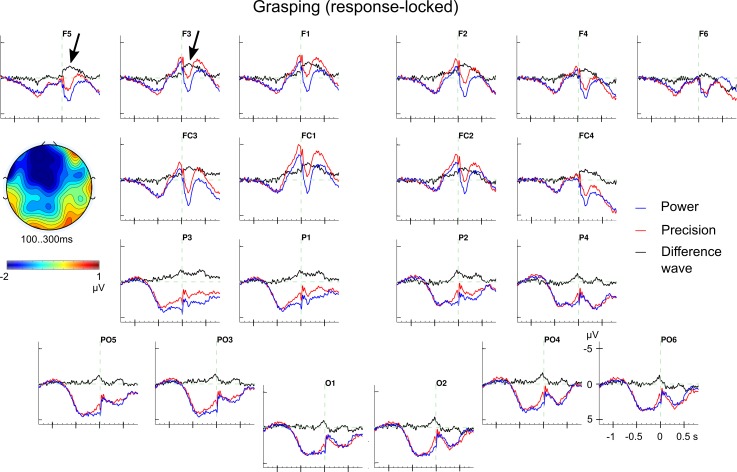
Response-locked ERP effect for grip types in grasping. Grand average ERPs for the grip types power (blue) and precision (red) and the difference wave (in black). Precision grips are associated with a more negative ERP than power grips in the left anterior ROI between 100 and 300 ms. The topographical map shows the left anterior scalp distribution of the grip type effect during movement execution.

Most importantly, in none of the explored time windows, the *noun concept × grip type* interaction was significant neither before (all *F*s_1,25_ < 1; all ns) nor after movement onset (all *F*s_1,25_ < 2.11; all *p*s > .159). Although significant *noun concept ×AP ×LR* interactions were found before movement onset in the time windows from -400 to 0 ms (*F*_1,25_ = 8.12; *p* < .01; η^*2*^ *=* 0.00013) and from -300 to -100 ms (*F*_1,25_ = 10.09; *p* < .01; η^*2*^ *=* 0.00014), none of the follow-up *t*-tests for separate ROIs yielded a significant main effect of *noun concept* (see S1 Table). (Note that there was also no effect involving any of the experimental factors between -100 & 0 ms) In contrast, after movement onset, there were significant interactions of *noun concept* with *AP* and *LR* (all *F*s_1,25_ > 5.25; all *p*s < .031; all η^*2*^ *>* 0.00002) as well as interactions of *grip type* with *AP* or *LR* (all *F*s_1,25_ > 4.53; all *p*s < .043; all η^*2*^ *>* 0.00017). However, follow-up *t*-tests failed to detect an effect of *noun concept* but yielded a main effect of *grip type* in the left-anterior ROI between 100 and 300 ms after movement onset (*t*_25_ = 2.08; *p* < .05; *Ω*^*2*^ *=* 0.0041). For the according ERPs, see Fig 7 and for the statistical values see S1 Table.

**Fig 8:**
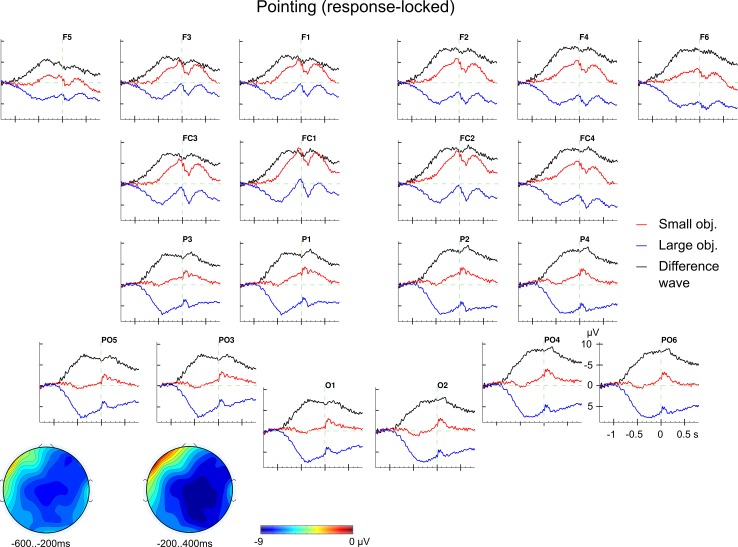
Response-locked ERP effect for object size in pointing. Grand average ERPs for small (red) and large objects (blue) together with the difference wave (in black). The effect of *object size* was broadly distributed and significant between 600 ms before and 400 ms after movement onset. The effect had a central maximum and did not interact with the ROI factors between -600 and -200 ms whereas it showed a right hemispheric dominance from -200 to 400 ms as indicated by the topographical maps (difference: small objects minus large objects).

The response-locked ERPs for the pointing block show a distinct pattern in comparison to the response-locked grasping ERPs (similar to the stimulus-locked analyses). Overall, there was a sustained negative shift for pointing at the small object compared with pointing at the large object (see Fig 8). Descriptively, this negative shift was broadly distributed with a centroparietal maximum and a right-hemispheric preponderance. The ERPs for pointing at small and large objects started to diverge, again descriptively, around 900 ms before movement onset. The typical components around movement onset are also pronounced (BP, PMP, MP & MEP; see above).

As these ERPs show a sustained difference, we decided to explore the ERPs in successive 100 ms time windows beginning 900 ms before movement onset and until 600 ms after movement onset. For each time window, we performed an ANOVA with the factors *noun concept*, *object size*, *LR* and *AP*. Critically, there was no significant interaction involving the two factors *noun concept* and *object size* in any of these time windows (all *F*s_1,25_ < 2.55; all ns). Interestingly, all time windows from -600 to 400 ms yielded a significant main effect of *object size* (all *F*s_1,25_ > 5.63; all *p*s < .026; all *Ω*^*2*^ *>* 0.0078) and all time windows from -200 to 600 ms yielded a significant interaction of *object size* and *LR* (all *F*s_1,25_ > 4.73; all *p*s < .04; all *Ω*^*2*^ *>* 0.0012; marginally significant for 300 to 400 ms, *F*_1,25_ = 3.56; *p* < .1; *Ω*^*2*^ *=* 0.0006). For full statistical details see S2 Table. No other effects showed a consistent pattern of results in these analyses.

Furthermore, the same four-way ANOVA was performed on the response-locked ERPs of the electrodes C3 and C4 for both blocks (grasping & pointing). There was, again, no interaction of *noun concept* and *grip type* (grasping) or *noun concept* and *object size* (pointing), nor was there any interaction involving the two factors (all *F*s_1,25_ < 2.35; all ns). Further statistical details are provided in the Supplementary information (S3 Table). Please refer also to S3–S30 Datasets for ERP values.

Overall in the grasping block, participants reacted faster to “large” nouns than to “small” nouns and grasped the large object earlier than the small object. Importantly, the ERPs showed an interactive processing of *conceptual noun information* and *grip type* between 100 and 200 ms after stimulus presentation. Incongruent noun-grip constellations elicited an increased posterior positivity compared with congruent constellations. There was also an increased negativity for large compared with small nouns which had a central maximum between 500 and 650 ms. In response-locked analyses, only *grip type* affected the ERP 100 to 300 ms after movement onset. Precision grips led to a greater negativity than power grips at left anterior electrodes. In the control block (pointing), participants initiated their responses also faster for large compared with small nouns. The ERPs showed a distinct pattern with a sustained negativity for pointing at the small vs. the large object (200–800 ms). Consistently, the response-locked ERP analyses yielded a sustained negativity for the small object (significant between -600 & 400 ms). Both ERP effects showed a centroparietal distribution with a right–hemispheric preponderance. Importantly, *noun concept* and *grip type* did not interact in any time window in the control block. For a summarised overview, see Table 4.

**Table 4:** Summary of main results for both blocks (grasping & pointing) and both analysis types (stimulus- & response-locked). The main statistical effects are given for reaction and movement times and mean ERP amplitude values. (ERP time windows are given in ms.)

	*Analyses*				
	*Stimulus-locked*			*Response-locked*	
	*RT*			*MT*	
*Block*	main effect **noun**			main effect **grip type**	
*Grasping*			*ERP*		
	100..200	500..650		100..300	
	interaction/	main effect		main effect	
	**congruency**	**noun**		**grip type**	
	(posterior)			(left anterior)	
	*RT*			***–***	
	main effect **noun**				
*Pointing*			*ERP*		
	200..800			-600..-200	-200..400
	main effect			main effect	main effect
	**object size**			**object size**	**object size**

## Discussion

This study investigated the neurophysiological processing of symbolically-coded, conceptual representations (action knowledge associated with noun representations) and concrete, motor commands (as part of grip type representations). The main ERP result was an early interaction of conceptual information and motor commands between 100 and 200 ms after word presentation at posterior electrodes which precedes the typical time window of semantic processing effects (N400). The findings support our hypothesis that action-related, conceptual information may gain priority for behavioural control if required by the task. That is, conceptual information may be processed earlier than in tasks with a more cognitive emphasis, cf. [61].

The behavioural data show that action-related, conceptual information affects response times (RTs) in the grasping block and also in the pointing block. Words that denote larger objects which require power grips led to shorter RTs than words that denote smaller objects which require precision grips. Interestingly, the RT effect of this conceptual information was independent of the task, i.e., it was found for grasping and for pointing. This result shows that word reading cannot only pre-activate further *perceptual* processes as often investigated in priming paradigms (i.e., a picture primes the perception of another picture) but it can also influence subsequent *motor* processes (i.e., a picture influences motor processes; cf. Vainio et al. [89] for grasp observation).

A behavioural grip type effect was only observed in movement times (MTs; grasping block) but not in any RT measure. Power grips were executed faster than precision grips, presumably reflecting the lower precision demands. This result is in accordance with a reduced velocity, reduced grip aperture and prolonged deceleration phase of precision grips compared with power grips [90,15,18,37]. Generally, these effects show that the manipulations were effective and suggest processing benefits when grasping needs less movement precision (power grips) but also that action-related, conceptual noun information can critically influence processing.

The behavioural results are generally in line with the two component model of voluntary movements [10,11,12]. In the present go/no-go task, words were relevant for the decision whether or not to execute a movement (in grasping & pointing). This should mainly influence the planning of the response and, accordingly, RTs showed a main effect of noun concept in the grasping and the pointing block but the MTs did not. In contrast, the various grip types had distinct movement requirements and, consistently, the MTs (i.e., durations) differed between power and precision grips but not the RTs. That is, the execution but not the planning component was apparently affected by grip types in support of the planning control distinction of the two component model.

As predicted, the stimulus-locked ERP analyses revealed an interaction of conceptual noun and grip type information between 100 and 200 ms after word onset in the grasping block. Incongruent combinations of noun and grip type elicited a more positive going ERP amplitude than congruent combination at posterior electrodes (cf. Fig 3). An early posterior positivity for incongruent human actions beginning 170 ms after picture presentation has been reported before (actions were semantically incongruent with the context or world knowledge [91]). However, this study is not strictly comparable to the present work as Proverbio and Riva [91] investigated postures of the whole body in more or less natural environments but the early ERP effect was interpreted to reflect visual semantic scene recognition (incl. the action).

Many ERP components in the time range of 100 to 200 ms, such as P1/N1/P2 were concerned with auditory or emotional processing. These components are oftentimes functionally related to (spatial or selective) attentional processing [92,93,94,95] but only the P1 component showed an increased positivity for attended stimuli ([96,94] posterior P2 effects are hardly investigated). More interestingly, there have been a number of studies that report an early influence of linguistic variables or language context on visual word recognition in such an early time range (without a direct connection to motor control processes). Sereno et al. [97] showed that already the P1 component (beginning 100 ms after stimulus presentation) was affected by word frequency, lexicality and regularity which is related to spelling (i.e., grapheme to phoneme conversion). Furthermore, Dambacher et al. [98,99] report early ERP amplitude modulations of word predictability (of word forms) that begin no later than 90 ms after stimulus presentation (other investigated variables include word length and position in the carrier sentence). Such effects reflect in influence of context information which serves as a basis for predictions, e.g., [100]. These studies suggest that the early congruency effect of nouns and required grip types may well reflect a modulation of lexical processing. Apparently the incongruity required additional processing capacity. Whether the congruency effect reflects facilitation for congruent or interference for incongruent conditions cannot be decided with the present data.

The early time window of the interaction is in line with ERP findings which showed an influence of language on the activity in the motor system in this early processing stage, e.g., [50,49,57]. More generally, the interaction supports the notion of domain interactions in line with functional interactions of verbal working memory and grasping [101,102,103,104]. The present interaction shows that symbolic, conceptual information can be accessible for action specification together with motor commands and earlier than found in the Amsel et al. study.

Importantly, the occurrence of this interaction (and the congruency effect) strongly suggests an integrated processing of symbolically coded information (nouns) and concrete motor commands (grip types). To the extent that the interaction reflects integrative processing of symbolic information and concrete, motor commands, i.e., a functional relation in processing, this finding supports embodiment views which argue for an integrative processing and against a strict separation of symbolic and sensory/motor information processing, e.g., [22,21]. Moreover, we believe that this main finding supports rather weak embodiment views [20] because access of conceptual information (living / non-living distinctions) have been found to precede the availability of graspability information in other tasks [61].

The early occurrence could be related to the fact that in the current task three potential responses (grips) might have been planned ahead which could be considered an easy task. However, response-locked ERPs did not show a difference in the Bereitschaftspotential (BP) among the experimental conditions. Hence, the preparatory effort as judged by the BP amplitude seems not to differ. This suggestion should be taken with care as the response-locked ERPs may have been noisier than the stimulus-locked ERPs, but the small response set in our study can be considered relatively easy in any case (in the sense of three grasping actions vs. many potential actions in unconstrained, every-day situations). That is, the assumption of pre-planning the three potential responses in the present set-up seems a plausible possibility. Importantly, the interaction of conceptual noun information and grip types in the grasping block (stimulus-locked) is suggestive of a functional relation between noun reading (i.e., associated action knowledge) and grasping behaviour.

It should also be noted that no interaction of conceptual information and grip types occurred in the pointing block (in any ERP analysis). It seems that conceptual information and motor commands are only processed together neurophysiologically, if a complex (manual) response is required and reading is directly related to the motor requirements (object manipulation). As grasping and pointing movements differ kinematically, e.g., [75] it is possible that the control processes for these movements also differ, cf. [31]. Therefore, pointing may be suboptimal as a control condition. Here, we can only state that a neurophysiological interaction of conceptual information and motor commands occurred for grasping movements. In the pointing block such an interaction could not be detected. It does, apparently, not suffice if the words in our study were task-relevant (as an imperative signal) in the pointing block.

Also, in the study of Amsel et al. [61], a cognitive judgement was strongly emphasised by the task, and action-related information (graspability) was processed only after about 350 ms. That is, the present neurophysiological interaction of conceptual information and grip type seems to be task-dependent [61] cf. also [62] and seems not to be related, alternatively, to the reaching component of the movement. More generally, the cognitive planning (or the anticipation of the action goal) seems to have an earlier influence in preparing and/or initiating a manual action, e.g., [16,17,53,18] when a complex (manual) response is required. That is, this present experiment constitutes preliminary evidence for the suggestion that the task requirements (e.g., complex action vs. cognitive evaluation) modulates the neurophysiological processing of behaviour.

Later in processing, conceptual information elicited a reduced negativity (500–650 ms) for “small” nouns compared to “large” nouns with a central scalp distribution. This reduced N400 may indicate that the actions associated with nouns for smaller objects may be specified in more motor detail (e.g., exactly two contact points, one for the index finger and one for the thumb) than actions associated with nouns for larger but still graspable objects since the nouns were matched for relevant linguistic features. Note that the words denoted objects, not actions, that is, the N400 effect might be related to the typical use of the objects. A more clearly defined representation of such action knowledge may be easier to access and could, thus, elicit a reduced N400 amplitude similar to words that elicited a reduced N400 amplitude compared to pseudo words, cf. [44,48].

In a related study, van Elk et al. [40] observed a similar N400 effect with a more negative amplitude for meaningful compared to meaningless manual actions (380–450 ms). Even though both studies are not directly comparable, it is noteworthy that in both cases the action-related N400 effects show a more anterior distribution than typically found [44,48] which might also be related to the involvement of motor processes originating, arguably, partly in frontal cortices (cf. [34] for anterior N400 effects during overt speech).

Interestingly, the present neurophysiological interaction of conceptual information and grip types (congruency effect) preceded the N400 effect for conceptual, noun information by about 300 ms. The time window of the interaction (100–200 ms) does not seem to capture the *onset* of an integrated processing step as no effects have been found in the time window 200–300 ms after word onset. Hence, the early interaction and the N400 effect for nouns seem to reflect two separate processes. For tasks as the one used here, it seems that motor commands for grasping can influence processing before language processes indexed by the N400 (i.e., lexical access or lexical integration, e.g., [44,45,48]).

Regarding the grip types, an ERP (main) effect was only observed in the response-locked analyses between 100 and 300 ms after movement initiation at left anterior electrodes (cf. Table 4). Precision grips elicited a more negative ERP amplitude than power grips (see Fig 7). Since response-locked ERP reflect motor-related brain processing, the grip type effect is suggested to index the control component of voluntary movements. Also, the occurrence of this grip type effect in the response-locked ERP corresponds to the behavioural grip type effect which occurred in the movement but not in the reaction times. Thus, this ERP grip type effect is in line with the distinction of precision and power grips as distinct grasping actions, e.g., [2,72].

Left anterior negativities (LAN) in a comparable time range have been reported before, albeit for lexical processing and in stimulus-locked ERPs. These LAN effects are commonly taken to reflect local structural processing (morphosyntax, to be precise); for example, the structural correspondence between a determiner and a noun’s syntactic gender [105,106] or between a verb and the sentence subject [107,108]. That is, these LAN effects are related to the (difficulty of) processing the structure of words in relation to their sequence. For the present ERP effect of grip type, it could be discussed whether it reflects, in analogy, the processing of some structural action aspects (of grasping) in a behavioural sequence (reading-reaching-grasping-lifting-placement in the present case). Such an “action LAN” for precision grips compared to power grips might reflect the *control* of the pre-shaping of the fingers while the reaching movement began. Or, alternatively, it could reflect the preparation of the final finger posture when grasping (enclosing the object). The latter could also be equivalent to accessing the action vocabulary [18], that is, activation of a grip type representation in the service of movement control (execution). Such common LAN effects for language and grasping would be suggestive of a domain general, neural sequence processing mechanism [109,110]. Interestingly, Proverbio et al. [58] reported a left anterior negativity in a similar time range (210–270 ms) albeit not for grasping but for the observation of manipulable objects (tools). These manipulable objects were compared with non-tools raising the interesting possibility that the LAN effect might reflect the processing of object-related action knowledge (e.g., a motor schema) with regard to a sequential action aspect. These theoretical considerations need, clearly, further experimental support.

The ERPs for the pointing block showed, surprisingly, a qualitatively different pattern of results. Here, a sustained slow negative shift for the small object (which would require a precision grip) relative to the large object was found in both, stimulus- and response-locked ERPs (see Figs 5 and 7). These effects were significant between 200 and 800 ms in the stimulus-locked and also between -600 and 400 ms in the response-locked analyses. Such a qualitatively difference in the neurophysiological brain response is in line with the argument that reaching and grasping represent distinct movement [72,73,74] but see [111].

These pointing ERP effects should not reflect the reaching component because reaching was the same for all objects. Instead, this slow shift could reflect an implicit effort for aiming at the object. Although participants were not instructed to point precisely, for example, at the centre of the objects, they may have done so. Imagined aiming with one specific finger, even though all fingers were physically extended when pointing, may have felt more difficult for a smaller “target area” (small object) than for a larger one (large object). Precise aiming can be assumed to involve planning and correct, that is, precise moving of the effector. Thus, it seems also plausible that a potential neurophysiological effect, the slow negative shift in the pointing block was found in the stimulus- and in the response-locked ERPs. Further research is needed to confirm these suggestions.

Although the present work focussed on neurophysiological correlates of the (integrative) processing of language and motor commands, no interaction was found in RTs or MTs but only in the ERPs for the grasping block. This leaves the question unanswered how neurophysiological activity relates to the behaviour. Generally, our behavioural and neurophysiological measures may differ in sensitivity. Other behavioural measures may be better suited such as kinematic parameters which can better capture the complexity of manual actions. Boulenger et al. [57], for example, observed interactive processing of language information and grasping in wrist acceleration but RTs did not show an effect. Similarly, Amsel et al. [61] reported no interaction of conceptual and graspability information in their RTs. Future studies may, thus, benefit from the simultaneous recording of ERPs and kinematic parameters [37].

To summarise, a neurophysiological interaction (congruency effect) of abstract conceptual noun information (i.e., action knowledge related to the nouns) and motor commands (as part of different grip types) have been reported in a grasp-and-lift task 100–200 ms after word presentation. This interaction supports our hypotheses that cortical processes for reading and (manual) action can become functionally related in an action situation, that is, if a complex motor response is required, in line with weak embodiment approaches. Furthermore, the interaction effect was followed by an N400 effect of conceptual noun information (500–650 ms, indicating lexical processing). The interaction suggests that motor commands for complex (manual) actions can influence processing at the same time as conceptual noun information and does not have to be processed *after* lexical access (cf. [61] where the task emphasised cognitive evaluation). The absence of a similar interaction effect in the pointing task tentatively suggests that the interaction reflects grasp-specific processing. That is, processing may depend on the nature of the task. In contrast, no interaction effect was obtained in the response-locked ERP analyses. Hence, the interaction in the stimulus-locked ERPs suggests that the interaction of conceptual and motor information occurs during the planning component of the movement but not during the execution phase. Consistently, a grip type effect was found in the response-locked ERP analyses which reflect brain activity that is related to motor execution processes; that is, the control of the movement (grasping) requirements. Generally, behavioural control may be task-specific. That is, action-related conceptual information seems to depend on the nature of the task and may gain processing priority (i.e., being processed earlier) when they pertain to a (complex) action requirement. Finally, the present results might ultimately help to better understand the diverse functions of the human hand ranging from physical tasks to social and verbal communication, and whether and when the language and the motor control systems interact in manual actions.

## Supporting Information

S1 Dataset Reaction time data; pointing block. (TXT)

S2 Dataset Reaction and movement time data; grasping block. (TXT)

S3 Dataset Mean ERP amplitude values for grasping; stimulus-locked 100..200 ms. (DAT)

S4 Dataset Mean ERP amplitude values for grasping; stimulus-locked 200..300 ms. (DAT)

S5 Dataset Mean ERP amplitude values for grasping; stimulus-locked 500..650 ms. (DAT)

S6 Dataset Mean ERP amplitude values for grasping; response-locked 0..300 ms. (DAT)

S7 Dataset Mean ERP amplitude values for grasping; response-locked 100..300 ms. (DAT)

S8 Dataset Mean ERP amplitude values for grasping; response-locked 200..600 ms. (DAT)

S9 Dataset Mean ERP amplitude values for grasping; response-locked -300..-100 ms. (DAT)

S10 Dataset Mean ERP amplitude values for grasping; response-locked 300..500 ms. (DAT)

S11 Dataset Mean ERP amplitude values for grasping; response-locked -400..0 ms. (DAT)

S12 Dataset Mean ERP amplitude values for pointing; stimulus-locked 100..200 ms. (DAT)

S13 Dataset Mean ERP amplitude values for pointing; stimulus-locked 200..800 ms. (DAT)

S14 Dataset Mean ERP amplitude values for pointing; stimulus-locked 500..650 ms. (DAT)

S15 Dataset Mean ERP amplitude values for pointing; response -locked 0..100 ms. (DAT)

S16 Dataset Mean ERP amplitude values for pointing; response-locked 0..400 ms. (DAT)

S17 Dataset Mean ERP amplitude values for pointing; response-locked -100..0 ms. (DAT)

S18 Dataset Mean ERP amplitude values for pointing; response-locked 100..200 ms. (DAT)

S19 Dataset Mean ERP amplitude values for pointing; response-locked -200..-100 ms. (DAT)

S20 Dataset Mean ERP amplitude values for pointing; response-locked 200..300 ms. (DAT)

S21 Dataset Mean ERP amplitude values for pointing; response-locked -300..-200 ms. (DAT)

S22 Dataset Mean ERP amplitude values for pointing; response-locked 300..400 ms. (DAT)

S23 Dataset Mean ERP amplitude values for pointing; response-locked -400..-300 ms. (DAT)

S24 Dataset Mean ERP amplitude values for pointing; response-locked 400..500 ms. (DAT)

S25 Dataset Mean ERP amplitude values for pointing; response-locked -500..-400 ms. (DAT)

S26 Dataset Mean ERP amplitude values for pointing; response-locked 500..600 ms. (DAT)

S27 Dataset Mean ERP amplitude values for pointing; response-locked -600..-500 ms. (DAT)

S28 Dataset Mean ERP amplitude values for pointing; response-locked -700..-600 ms. (DAT)

S29 Dataset Mean ERP amplitude values for pointing; response-locked -800..-700 ms. (DAT)

S30 Dataset Mean ERP amplitude values for pointing; response-locked -900..-800 ms. (DAT)

S1 Fig Grasping (stimulus-locked). Stimulus-locked, grand average ERPs for the four conditions (*noun concept* × *grip type*) in the grasping block to illustrate further the interaction of both factors between 100 and 200 ms; cf. Fig 3.
(TIF)

S1 Table Grasping. Response-locked ERP results (*t*-tests) for mean amplitudes of various time windows before and after movement onset. *T*-values for 25 degrees of freedom. *T*-tests for only Ant/Pos and only for Left/Right were supported by grip type × AP and grip type × LR, respectively. Significant effects are given in boldface.
(DOCX)

S2 Table Pointing. Response-locked ERP results (ANOVA: noun × grip × AP × LR) for mean amplitudes of successive 100 ms time windows (zero indicates response button release). *F* values for 1,25 degrees of freedom. Non reported interactions yielded no significant effects (and no tendencies). Significant effects are given in boldface.
(DOCX)

S3 Table Both blocks. Response-locked ERP results for mean amplitudes at the electrodes C3 (left) and C4 (right hemisphere). *F*- and *t-*values for 1,25 and 25 degrees of freedom, respectively. Significant effects are given in boldface.
(DOCX)
