# Supplementary figures and images for: Action Priority: Early Neurophysiological Interaction of Conceptual and Motor Representations
Source: PLoS One. 2016 Dec 14;11(12):e0165882. doi: 10.1371/journal.pone.0165882 (PMC5156427; doi:10.1371/journal.pone.0165882)

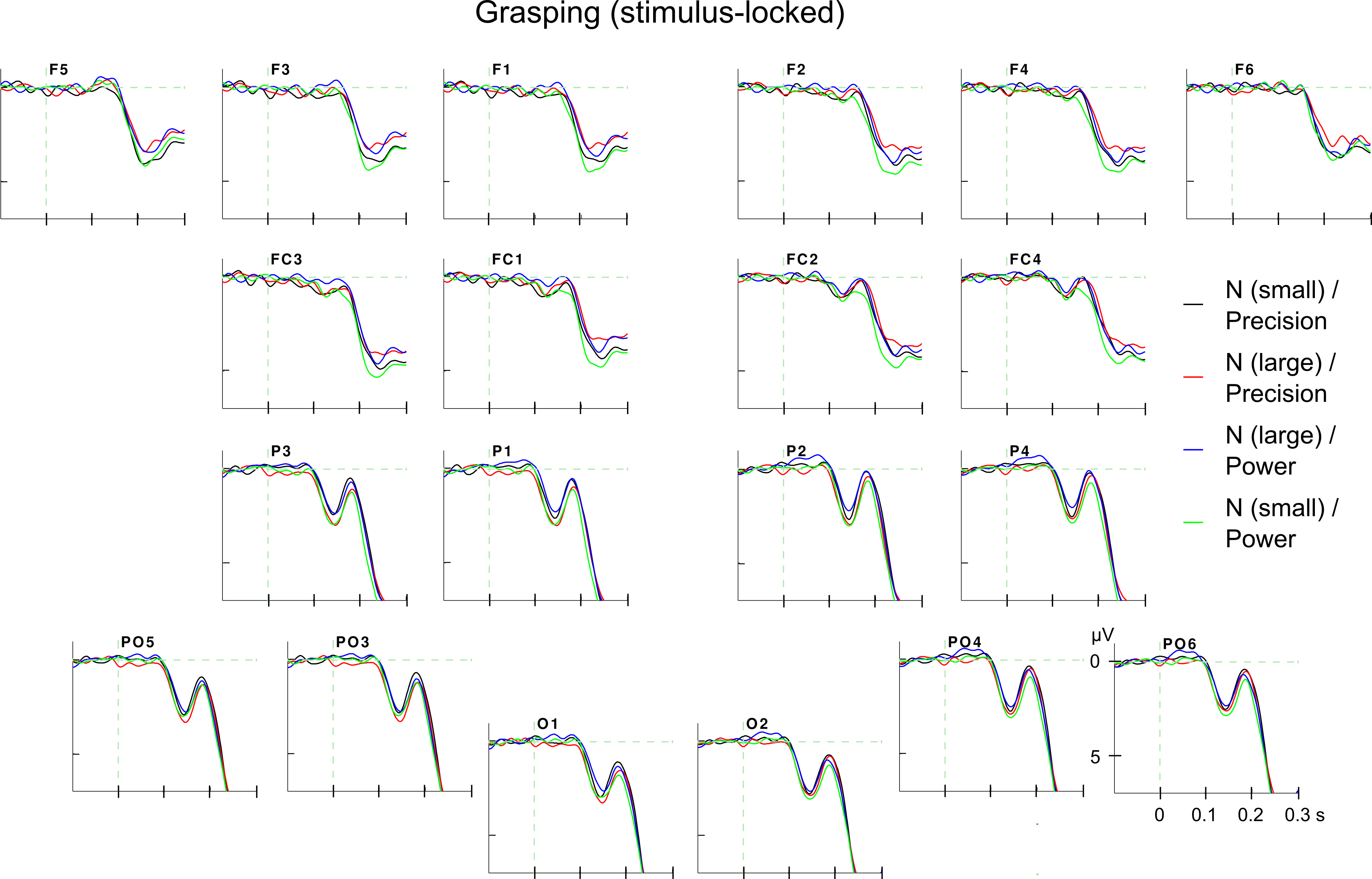

Supplement: S1 Fig — Stimulus-locked, grand average ERPs for the four conditions (noun concept × grip type) in the grasping block to illustrate further the interaction of both factors between 100 and 200 ms; cf. Fig 3. (TIF) [file pone.0165882.s031.tif]
