# Supplementary material for: Action Priority: Early Neurophysiological Interaction of Conceptual and Motor Representations
Source: PLoS One. 2016 Dec 14;11(12):e0165882. doi: 10.1371/journal.pone.0165882 (PMC5156427; doi:10.1371/journal.pone.0165882)
Supplement: S1 Table — Response-locked ERP results (t-tests) for mean amplitudes of various time windows before and after movement onset. T-values for 25 degrees of freedom. T-tests for only Ant/Pos and only for Left/Right were supported by grip type × AP and grip type × LR, respectively. Significant effects are given in boldface. (DOCX) [file pone.0165882.s032.docx]

**S1 Table. Grasping.** Response-locked ERP results (*t*-tests) for mean amplitudes of various time windows before and after movement onset. *T*-values for 25 degrees of freedom. *T*-tests for only Ant/Pos and only for Left/Right were supported by grip type × AP and grip type × LR, respectively. Significant effects are given in boldface.

| *Time window* | | | | | | |
| --- | --- | --- | --- | --- | --- | --- |
| (before after movement onset) | | | | | | |
| *ROI/effect* | *-400..*  *0* | *-300..*  *-100* | *0..*  *300* | *100..*  *300* | *200..*  *500* | *300..*  *500* |
| Noun/AL*, t*  *p* | 0.30  .7696 | 0.18  .8624 | 0.72  .4785 | 0.72  .4791 | 0.67  .5118 | 0.71  .4820 |
| Noun/AR*, t*  *p* | 0.26  .7940 | 0.50  .6199 | 0.51  .6124 | 0.40  .6894 | 0.38  .7070 | 0.39  .6966 |
| Noun/PL*, t*  *p* | 0.65  .5221 | 0.83  .4167 | 0.04  .9672 | 0.11  .9154 | 0.39  .7001 | 0.38  .7095 |
| Noun/PR*, t*  *p* | 0.36  .7255 | 0.65  .5205 | 0.58  .5693 | 0.37  .7133 | 0.89  .8933 | 0.08  .9397 |
| Grip/AL*, t*  *p*  *Ω^2^* |  |  | Ant:  1.88 | **2.08**  .0478  .0010 | Left:  0.71 0.68 | |
| Grip/AR*, t*  *p* |  |  | .0712 | 1.13  .2679 | .4816 | .5009 |
| Grip/PL*, t*  *p* |  |  | Post:  0.16 | 0.10  .9198 | Right:  0.18 0.14 | |
| Grip/PR*, t*  *p* |  |  | .8727 | 0.24  .8093 | .8573 | .8929 |

Note: Ant – anterior; Post – posterior; AL – anterior left; AR – anterior right; PL – posterior left; PR – posterior right.
