# Supplementary material for: Action Priority: Early Neurophysiological Interaction of Conceptual and Motor Representations
Source: PLoS One. 2016 Dec 14;11(12):e0165882. doi: 10.1371/journal.pone.0165882 (PMC5156427; doi:10.1371/journal.pone.0165882)
Supplement: S2 Table — Response-locked ERP results (ANOVA: noun × grip × AP × LR) for mean amplitudes of successive 100 ms time windows (zero indicates response button release). F values for 1,25 degrees of freedom. Non reported interactions yielded no significant effects (and no tendencies). Significant effects are given in boldface. (DOCX) [file pone.0165882.s033.docx]

**S2 Table. Pointing.** Response-locked ERP results (ANOVA: noun × grip × AP × LR) for mean amplitudes of successive 100 ms time windows (zero indicates response button release). *F* values for 1,25 degrees of freedom. Non reported interactions yielded no significant effects (and no tendencies). Significant effects are given in boldface.

| *Time window* | | | | | | | | | | | | | | | |
| --- | --- | --- | --- | --- | --- | --- | --- | --- | --- | --- | --- | --- | --- | --- | --- |
| *Effect* | *-900..*  *-800* | *-800..*  *-700* | *-700..*  *-600* | *-600..*  *-500* | *-500..*  *-400* | *-400..*  *-300* | *-300..*  *-200* | *-200..*  *-100* | *-100..*  *0* | *0..*  *100* | *100..*  *200* | *200..*  *300* | *300..*  *400* | *400..*  *500* | *500..*  *600* |
| Noun, *F*  *p*  *Ω^2^* | 0.61 | 0.01 | 2.92 | 0.28 | 0.61 | 0.24 | 2.76 | 3.06  .0926  .0016 | 0.16 | 0.20 | 0.14 | 0.71 | 0.96 | 0.33 | 0.50 |
| Grip, *F*  *p*  *Ω^2^* | **11.23**  .0026  .0373 | 2.87 | 0.09 | **5.63**  .0256  .0079 | **11.00**  .0028  .0357 | **14.64**  .0008  .0644 | **11.98**  .0019  .0427 | **9.91**  .0042  .0285 | **10.67**  .0032  .0334 | **8.79**  .0066  .0220 | **14.34**  .0009  .0617 | **10.83**  .0030  .0345 | **7.45**  .0114  .0152 | 3.79  .0628  .0029 | 1.99 |
| Grip × AP, *F*  *p*  *Ω^2^* | 2.68 | 1.12 | 0.09 | 0.15 | 0.01 | 0.55 | 1.11 | 1.66 | 1.71 | **9.85**  .0043  .0072 | **12.44**  .0017  .0120 | 2.62 | 1.74 | 1.91 | 1.22 |
| Grip × LR, *F*  *p*  *Ω^2^* | 2.92 | 3.21  .0854  .0005 | 0.73 | 0.09 | 0.01 | 0.54 | 2.76 | **7.94**  .0093  .0044 | **10.38**  .0035  .0081 | **14.72**  .0008  .0171 | **15.65**  .0006  .0195 | **4.74**  .0391  .0013 | 3.56  .0709  .0006 | **5.15**  .0322  .0016 | **6.30**  .0189  .0026 |
| Grip × AP × LR, *F*  *p* | 2.82 | 0.02 | 1.60 | **5.29**  .0301 ^a^ | **6.15**  .0203 ^a^ | 2.81 | 1.03 | 1.22 | 0.93 | 0.64 | 0.72 | 2.04  .0990 | 1.59 | 1.36 | 0.10 |

^a^ For full within-subjects designs with three or more factors, there is no general agreement how *Ω^2^* should be calculated reliably. As a surrogate generalised η^2^ is reported here.
