# Supplementary material for: Action Priority: Early Neurophysiological Interaction of Conceptual and Motor Representations
Source: PLoS One. 2016 Dec 14;11(12):e0165882. doi: 10.1371/journal.pone.0165882 (PMC5156427; doi:10.1371/journal.pone.0165882)
Supplement: S3 Table — Response-locked ERP results for mean amplitudes at the electrodes C3 (left) and C4 (right hemisphere). F- and t-values for 1,25 and 25 degrees of freedom, respectively. Significant effects are given in boldface. (DOCX) [file pone.0165882.s034.docx]

**S3 Table. Both blocks.** Response-locked ERP results for mean amplitudes at the electrodes C3 (left) and C4 (right hemisphere). *F*- and *t-*values for 1,25 and 25 degrees of freedom, respectively. Significant effects are given in boldface.

|  | *Time window* | | | | | |
| --- | --- | --- | --- | --- | --- | --- |
|  | | Grasping | | | | Pointing |
| *Effect* | | *0..*  *300* | *200..*  *600* | | *300..*  *500* | *0..*  *400* |
| Noun, *F*  *p* | | 0.37  .5497 | 0.18  .6792 | | 0.26  .6139 | 0.41  .5256 |
| Grip, *F*  *p*  *Ω^2^* | | 1.61  .2168 | 0.58  .4548 | | 0.80  .3793 | **12.47**  .0016  .0500 |
| Noun × Grip, *F*  *P* | | 1.32  .2608 | 1.02  .3215 | | 0.52  .4775 | 2.35  .1381 |
| Grip × LR, *F*  *p*  *Ω^2^* | | 2.48  .1282 | **6.24**  .0194  .0027 | | **5.71**  .0248  .0022 | 2.02  .1676 |
| Noun × Grip × LR, *F*  *p* | | 2.20  .1504 | 1.84  .1867 | | 2.11  .1585 | 1.04  .3177 |
| Grip type *t*-test | |  | | Left: | |  |
| *t*  *p* | |  | | 1.15  .2625 | 1.27  .2164 |  |
| Grip type *t*-test | |  | | Right: | |  |
| *t*  *p* | |  | | 0.38  .7078 | 0.52  .6050 |  |
